# Peri-Tumoural Lipid Composition and Hypoxia for Early Immune Response to Neoadjuvant Chemotherapy in Breast Cancer

**DOI:** 10.3390/ijms25179303

**Published:** 2024-08-28

**Authors:** Sai Man Cheung, Kwok-Shing Chan, Nicholas Senn, Ehab Husain, Ravi Sharma, Trevor McGoldrick, Tanja Gagliardi, Yazan Masannat, Jiabao He

**Affiliations:** 1 Newcastle Magnetic Resonance Centre, Translational and Clinical Research Institute, Faculty of Medical Sciences, Newcastle University, Newcastle upon Tyne NE4 5PL, UK; jiabao.he@newcastle.ac.uk; 2 Athinoula A. Martinos Center for Biomedical Imaging, Department of Radiology, Massachusetts General Hospital, Charlestown, MA 02129, USA; 3 Harvard Medical School, Boston, MA 02115, USA; 4 Institute of Medical Sciences, School of Medicine, Medical Sciences and Nutrition, University of Aberdeen, Aberdeen AB25 2ZD, UK; 5 Department of Pathology, Aberdeen Royal Infirmary, Aberdeen AB25 2ZN, UK; 6 Department of Oncology, Aberdeen Royal Infirmary, Aberdeen AB25 2ZN, UK; 7 Department of Radiology, Royal Marsden Hospital, London SW3 6JJ, UK; 8 Breast Unit, Broomfield Hospital, Mid and South Essex NHS Foundation Trust, Chelmsford CM1 7ET, UK

**Keywords:** breast cancer, chemotherapy, hypoxia, inflammation, lipid composition, tumour-associated macrophages

## Abstract

The deregulation of monounsaturated, polyunsaturated, and saturated fatty acids (MUFAs, PUFAs, SFAs) from de novo synthesis and hypoxia are central metabolic features of breast tumour. Early response markers for neoadjuvant chemotherapy (NACT) are critical for stratified treatment for patients with breast cancer, and restoration of lipid metabolism and normoxia might precede observable structural change. In this study, we hypothesised that peri-tumoural lipid composition and hypoxia might be predictive and early response markers in patients with breast cancer undergoing NACT. Female patients with breast cancer were scanned on a 3T clinical MRI scanner at baseline and Cycle1, with acquisition of lipid composition maps of MUFAs, PUFAs, and SFAs, and hypoxia maps of effective transverse relaxation rate R2*. The percentage change in lipid composition and hypoxia at Cycle1 was calculated with reference to baseline. Tumour-associated macrophages were analysed based on immunostaining of CD163 from biopsy and resection, with the percentage change in the resected tumour calculated across the entire NACT. We found no significant difference in lipid composition and R2* between good and poor responders at baseline and Cycle1; however, the correlation between the percentage change in MUFAs and PUFAs against CD163 suggested the modulation in lipids with altered immune response might support the development of targeted therapies.

## 1. Introduction

Neoadjuvant chemotherapy (NACT) is increasingly used in breast cancer to facilitate breast conservation therapy [1] and avoid axillary clearance [2], and to improve disease-free survival [3,4] and conversion to surgery in patients with initially local advanced inoperable tumours [1]. Early identification of responders and non-responders remains critical for optimal patient care, since early identification supports stratified treatment and minimises the unnecessary potential adverse effects in non-responding patients [5]. An alteration in breast tumour metabolism precedes observable structural change [6], whereas current imaging markers of change in tumour size are inadequate. Altered lipid metabolism is shown to facilitate tumour proliferation [7], and treatment-induced normalisation in lipid composition might serve as a key metabolic marker in early immune response to NACT. Elevated hypoxia from aerobic glycolysis suggests advanced breast cancer [8], and although a change in perfusion fraction from intravoxel incoherent motion might show potential for early response [9], hypoxia-induced modulation of imaging marker effective transverse relaxation rate R2* showed robust correlation with hypoxia-inducible factor-1α [10], an endogenous hypoxia regulator critical for breast cancer metastasis [11].

The altered lipid composition from de novo synthesis is a central metabolic feature of breast tumour and is associated with tumour aggressiveness [7]. Breast cancer extracts exogenous fatty acids for phospholipids in membrane synthesis to sustain rapid cell growth [12]. Meanwhile, endogenous fatty acids are exported to avoid lipotoxicity, leading to a deregulation of monounsaturated, polyunsaturated, and saturated fatty acids (MUFAs, PUFAs, SFAs) in peri-tumoural adipose tissue [13]. The deregulation of lipid metabolism has been observed in breast adipose of BRCA1/2 genetic mutation carriers and has been proposed as a precursor of tumour initiation in the breast [14]. The decrease in peri-tumoural MUFAs was associated with tumour cellular differentiation and inversely correlated with Ki-67 at the advancing edge of the tumour [13]. Pro-inflammatory mediators, derived from de novo lipogenesis of MUFAs and PUFAs, fuel fatty acid oxidation in lipid scavenging receptors, including CD163 on tumour-associated macrophages (TAMs) [15], leading to polarisation in pro-tumoural M2 macrophages and continual immune modulation during tumour progression [16]. It has been shown that there is a strong link between peri-tumoural lipid composition and tumour immune function in patients with breast cancer [17]. NACT induces a modulation in immune function [18,19], and the shift in immune response might impact de novo lipogenesis after treatment as an early sign of tumour regression in response to NACT.

Peri-tumoural oedema consequent to inflammation generates extra osmotic pressure, restricting microcirculation and leading to hypoxia, and is associated with worse recurrence-free survival in triple-negative breast cancer [20,21,22]. The increase in oxygen consumption in TAMs increases the rate of glycolysis and activates hypoxia-inducible factor, creating a positive feedback loop linking immune function to hypoxia [23]. At the acute phase of NACT, an increase in MUFA-derived phosphatidylcholine and oxidised phospholipids predispose cancer cell apoptosis [24], triggering a further release of pro-inflammatory mediators in the tumour microenvironment [25]. A proximity between clusters of CD163 colonies and an elevation in CD163 adjacent to tumour are independent predictors of poor prognosis in patients after NACT [26]. Hence, the restoration of peri-tumoural lipid composition and normoxia might be central markers for early identification of responders. Novel chemical shift-encoded imaging (CSEI) provides a rapid and accurate map of lipid composition in the whole breast, and deploys a model lipid spectrum to estimate the number of double bonds in triglycerides to derive the individual lipid constituents, including MUFAs, PUFAs, and SFAs, while enabling the estimation of tissue hypoxia through R2* [13]. The CSEI method has been optimised for both ex vivo and in vivo breast imaging with demonstrable clinical value [13,17], and may serve response monitoring of NACT for stratified treatment.

**Figure 1:**
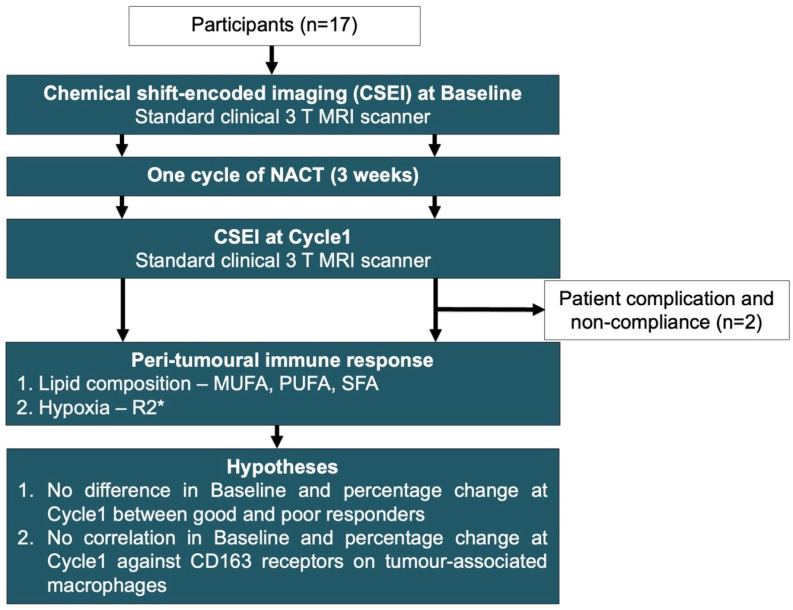
Lipid composition and hypoxia images were acquired before and after one cycle of neoadjuvant chemotherapy (baseline and Cycle1). The quantification of the number of double bonds was conducted through a simplified triglyceride model to derive monounsaturated, polyunsaturated, and saturated fatty acids (MUFAs, PUFAs, SFAs). Hypoxia was assessed via effective transverse relaxation rate R2*. The differences in baseline and percentage change at Cycle1 between good and poor responders were examined, with patients grouped according to the Miller–Payne system for pathological response (research question 1). The baseline and percentage change in lipid composition and R2* were correlated against CD163 in the biopsy and the percentage change in the resected tumour, respectively, in all participants (research question 2).

We therefore hypothesise that peri-tumoural lipid composition coupled with tissue hypoxia from CSEI may differentiate good responders from poor responders at baseline and after one cycle of NACT. We further hypothesise that changes in lipid composition and hypoxia modulate immune function, and in turn provide sensitive predictive, early response, and prognostic markers to NACT. We conducted a prospective, longitudinal study in female patients with breast cancer to probe changes in peri-tumoural lipid composition and hypoxia during NACT (Figure 1).

## 2. Results

### 2.1. Baseline Characteristics of the Study Patients

The patient demography is shown in Table 1. Among the 15 patients at Cycle1, 8 patients were poor responders and 7 patients were good responders. There was no significant difference in age and tumour size at baseline between poor and good responders.

### 2.2. Difference between Good and Poor Responders

**Figure 2:**
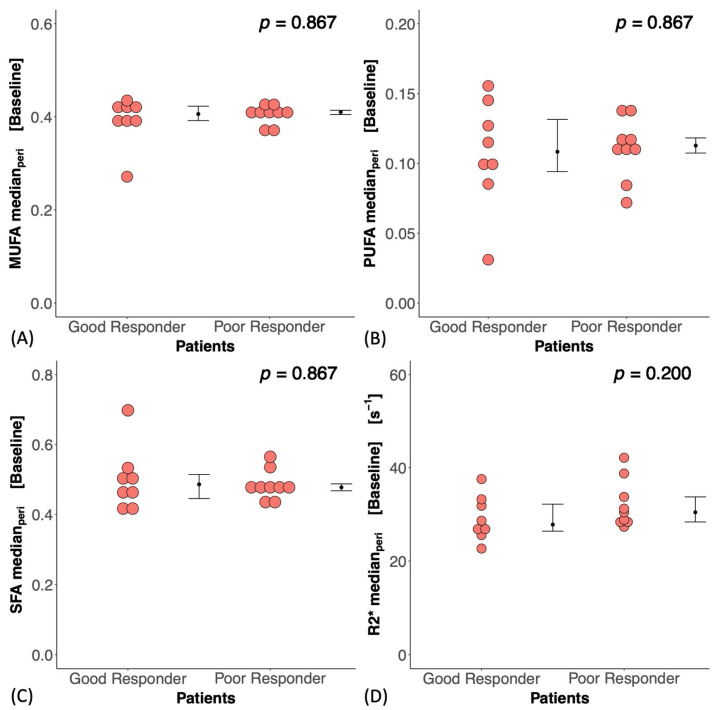
Peri-tumoural (**A**) monounsaturated fatty acids (MUFAs), (**B**) polyunsaturated fatty acids (PUFAs), (**C**) saturated fatty acids (SFAs), and (**D**) R2* between good and poor responders before neoadjuvant chemotherapy (baseline). There was no significant difference in baseline lipid composition or R2* between good and poor responders for all comparisons. Each dot represents the fraction of lipid composition or R2* in an individual patient. The error bar represents the median (IQR), and *p*-values are shown in the upper right corner.

**Figure 3:**
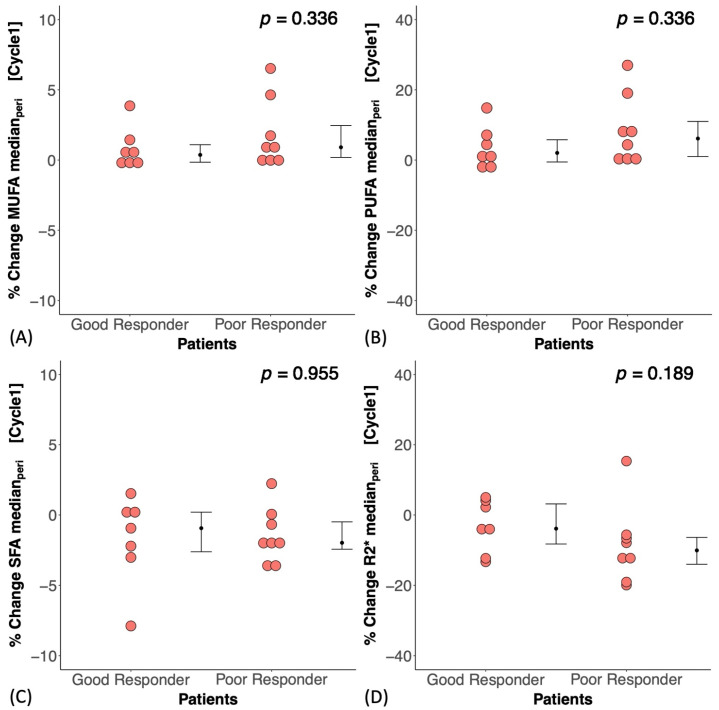
The percentage change in peri-tumoural (**A**) monounsaturated fatty acids (MUFAs), (**B**) polyunsaturated fatty acids (PUFAs), (**C**) saturated fatty acids (SFAs), and (**D**) R2* between good and poor responders after one cycle of neoadjuvant chemotherapy (Cycle1). There was no significant difference in the percentage change in lipid composition or in R2* between good and poor responders for all comparisons. Each dot represents the percentage change of lipid composition or R2* in an individual patient. The error bar represents the median (IQR), and *p*-values are shown in the upper right corner.

**Figure 4:**
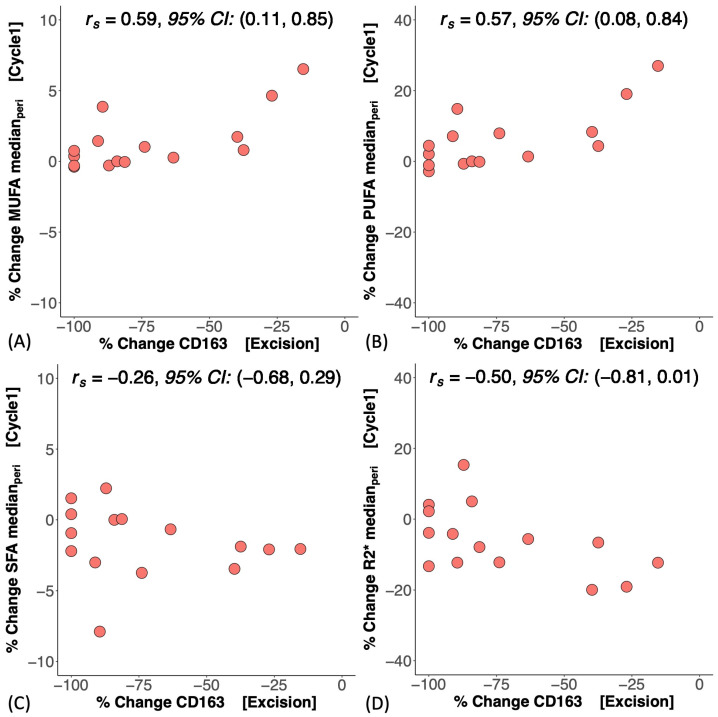
The correlations between the percentage change in peri-tumoural (**A**) monounsaturated fatty acids (MUFAs), (**B**) polyunsaturated fatty acids (PUFAs), (**C**) saturated fatty acids (SFAs), and (**D**) R2* after one cycle of neoadjuvant chemotherapy (Cycle1) and the percentage change in CD163 at excision are shown in scatter plots. Spearman’s rank correlation coefficient (*r_s_*) was used for correlation analysis, and the respective 95% confidence intervals (95% CI) are shown in each plot.

**Figure 5:**
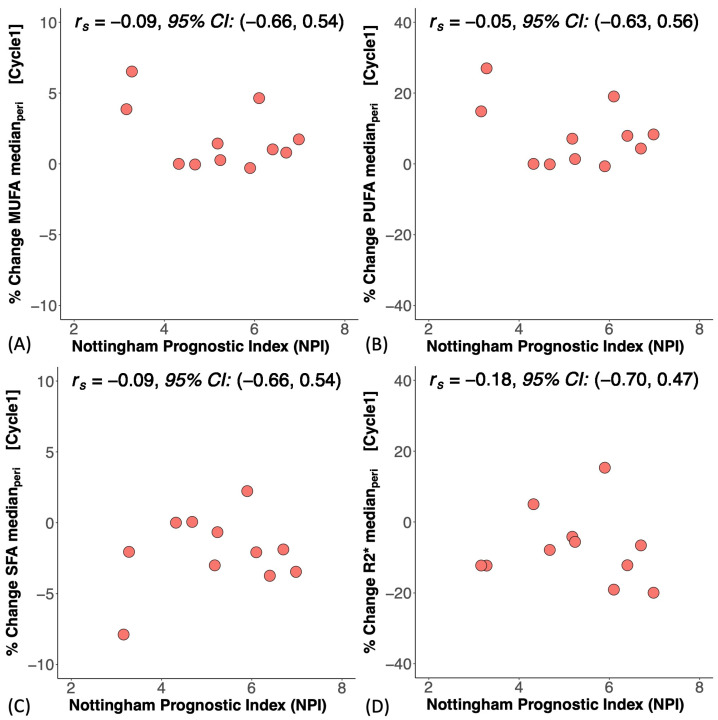
The correlations between the percentage change in peri-tumoural (**A**) monounsaturated fatty acids (MUFAs), (**B**) polyunsaturated fatty acids (PUFAs), (**C**) saturated fatty acids (SFAs), and (**D**) R2* after one cycle of neoadjuvant chemotherapy (Cycle1) and the Nottingham Prognostic Index (NPI) at excision are shown in scatter plots. Spearman’s rank correlation coefficient (*r_s_*) was used for correlation analysis, and the respective 95% confidence intervals (95% CI) are shown in each plot.

At baseline, there was no significant difference in MUFAs (*p* = 0.867), PUFAs (*p* = 0.867), SFAs (*p* = 0.867), or R2* (*p* = 0.200) between good and poor responders (Figure 2A–D). There was no correlation between MUFAs, PUFAs, SFAs, or R2* and CD163 in the core biopsy (Supplementary Figure S1A–D). At Cycle1, there was no significant difference in the percentage change in MUFAs (*p* = 0.336), PUFAs (*p* = 0.336), SFAs (*p* = 0.955), or R2* (*p* = 0.189) between good and poor responders (Figure 3A–D, Table 2). There was a significant correlation between the percentage change in MUFAs and the percentage change in CD163 (*r_s_* = 0.59, *p* = 0.021, Figure 4A, Table 2) in the entire cohort. There was a significant correlation between the percentage change in PUFAs and the percentage change in CD163 (*r_s_* = 0.57, *p* = 0.027, Figure 4B, Table 2). There was no correlation between the percentage change in SFAs (*r_s_* = −0.26, *p* = 0.354, Figure 4C, Table 2) or R2* (*r_s_* = −0.50, *p* = 0.056, Figure 4D, Table 2) and the percentage change in CD163. There was no significant correlation between the percentage change in MUFAs (*r_s_* = −0.09, *p* = 0.790), PUFAs (*r_s_* = −0.05, *p* = 0.873), SFAs (*r_s_* = −0.09, *p* = 0.790), or R2* (*r_s_* = −0.18, *p* = 0.593) and the Nottingham Prognostic Index (Figure 5, Table 2).

### 2.3. Difference between Baseline and Cycle1

**Figure 6:**
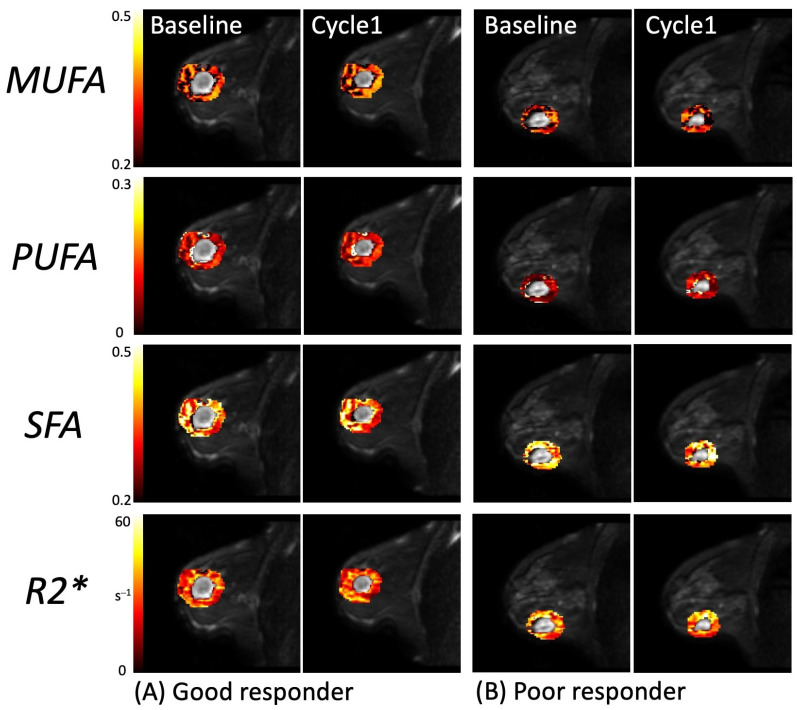
Quantitative maps of peri-tumoural monounsaturated fatty acids (MUFAs), polyunsaturated fatty acids (PUFAs), saturated fatty acids (SFAs), and R2* from a typical (**A**) good responder and (**B**) poor responder at baseline and Cycle1 of neoadjuvant chemotherapy (overlaid on anatomical images).

**Figure 7:**
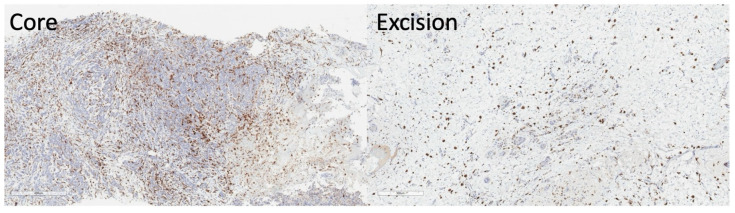
The reduction in CD163 in the resected tumour from the core biopsy on immunostaining slides from a typical patient undergoing neoadjuvant chemotherapy (NACT). The score for CD163 receptors on tumour-associated macrophages (TAMs) was 848.1 cells μm^−2^ in the core biopsy (**left**) and 134.9 cells μm^−2^ in the resected tumour (**right**). CD163+ TAMs, brown; Magnification, ×20.

Between baseline and Cycle1, in the entire cohort, there was a significantly lower R2* (*p* = 0.018) at Cycle1 compared to baseline (Supplementary Figure S2D and Supplementary Table S1). There was no significant difference at the borderline in MUFAs (*p* = 0.055), PUFAs (*p* = 0.064), or SFAs (*p* = 0.055) at Cycle1 compared to baseline (Supplementary Figure S2A–C and Supplementary Table S1). A direct comparison of the difference in response between good and poor responders at baseline and Cycle1 is shown in Figure S3. The quantitative maps of MUFAs, PUFAs, SFAs, and R2* from a typical good and poor responder at baseline and Cycle1 are shown in Figure 6. The reduction in CD163 in the resected tumour from the core biopsy on immunostaining slides from a typical patient is shown in Figure 7.

## 3. Discussion

In this study, we investigated the potential of peri-tumoural lipid composition and hypoxia as predictive, early response, and prognostic markers in patients with breast cancer undergoing NACT using MUFAs, PUFAs, SFAs, and R2* at baseline and Cycle1. We found no significant difference in lipid composition and R2* between good and poor responders at baseline or after the first cycle of NACT. There was a correlation between the percentage change in MUFAs and PUFAs and the percentage change in CD163, but not in SFAs or R2*. There was no correlation between lipid composition and R2* at baseline and CD163 in the core biopsy. There was no correlation between the percentage change in lipid composition and R2* and the Nottingham Prognostic Index. In the entire cohort, there was a significant decrease in R2* after the first treatment cycle; however, there was no significant difference at the borderline in MUFAs, PUFAs, or SFAs.

There was no significant difference in lipid composition or hypoxia in the peri-tumoural region at baseline between good and poor responders, and the results showed that lipid composition and hypoxia might not be the primary focus for predicting NACT responsiveness in breast cancer. A high lipid content in the tumour microenvironment accelerated the development of breast cancer cells in vitro and elevated metastatic capacity [27]; however, the absence of a difference in the degree of lipid deregulation between good and poor responders might be due to the complex interplay between lipid metabolism and individual immune response. Hypoxia is prevalent in the tumour microenvironment [23]; however, hypoxia might be the end product but not the upstream driver of progression in patients with breast cancer. There was also no significant difference in the percentage change in lipid composition and hypoxia after one cycle, and the results showed that lipid composition and hypoxia might not be the primary focus for early response to NACT. A reduction in lipid proteins, including fatty acid binding protein 4, which regulates inflammatory and metabolic processes in adipocytes and TAMs, led to reduced metastasis and enhanced sensitivity to chemotherapy in breast cancer xenografts [27]. However, the net change in the magnitude of individual lipid constituent might not be the upstream driver of response in NACT between responder groups in the patient population. The post-treatment release of inflammatory cytokines and modulation of hypoxia-inducible factor in the peri-tumoural region might lead to immune homeostasis at the tumour boundary and the surrounding microenvironment and confound hypoxic response in NACT between responder groups [28].

There was a strong correlation between the percentage change in MUFAs and PUFAs after one cycle of NACT and the percentage change in CD163, indicating that an increased bioavailability of MUFAs and PUFAs was associated with a sustained immune response of the tumour, although not a predictor of overall treatment response [16]. MUFAs and PUFAs, with their unique double-bond position, acted as active signalling agents to cascade immune function [29]. Increased lipolysis of exogenous peri-tumoural lipid droplets into unsaturated fatty acids not only supports anti-tumoural M1 macrophages [30,31] but also enhances fatty acid oxidation critical for activation of pro-tumoural M2 macrophages [32,33], including CD163 [26], an immunosuppressive phenotype promoting immune evasion [16]. The high expression of CD163 at baseline and the decrease in CD163 across the entire cohort after treatment indicates that NACT arrested the adaptive immune response during treatment [34]. The results showed that MUFAs and PUFAs in vivo are important surrogates for CD163-mediated M2 macrophage polarisation and protumourigenic effects during cell adaptation for survival [27], with important clinical relevance since CD163 remained high in metastases [35]. The absence of correlation between SFAs and CD163 might be due to SFAs, as a primary energy store, being less active in biological signalling during immune response [36]. The absence of correlation between hypoxia and CD163 indicates that hypoxia, while regulating inflammation and immune response, impacts specific antibodies in TAMs [23]. The absence of correlations in MUFAs, PUFAs, SFAs, and hypoxia at baseline against CD163 in the core biopsy was potentially due to natural variability in the patient population. The lack of significant correlation between lipid composition and hypoxia and the Nottingham Prognostic Index indicates that early treatment-induced normalisation in lipid composition and hypoxia, although potential markers of immune response might not be sensitive to the long-term survival offered by pathology.

In the entire cohort, there was a significantly lower R2* after the first treatment cycle, showing a mitigation in hypoxia. A restoration of peri-tumoural physiological angiogenesis improves tissue hypoxia, leading to a decrease in effective transverse relaxation rate R2* [37]. There was no significant difference at the borderline in MUFAs, PUFAs, or SFAs after the first treatment cycle, showing that a complete treatment-induced lipid normalisation might not occur at an early phase of NACT. However, the insignificant difference at the borderline might show a difference in response in a larger cohort, even though it did not reach the statistical threshold as a primary force in this study.

To the best of our knowledge, this is the first study using combined peri-tumoural lipid composition and hypoxia maps for response stratification after one cycle of NACT. The results suggest early treatment-induced modulation on MUFAs and PUFAs was correlated with the immune response of CD163-mediated macrophages upon completion of NACT, with unique prognostic value [26] and the potential to support the development of novel immunotherapy agents. Lipid metabolic reprogramming of TAMs is increasingly exploited in targeted therapies, including a vismodegib trial in breast cancer cell lines [38], to increase the ratio of M1/M2-like TAMs and enhance TAM phagocytic activity [39]. Non-invasive imaging markers are deployed in the neoadjuvant setting to account for the heterogeneity of the tumour microenvironment while avoiding partial sampling error and disrupting the integrity of the tissue being tested. Our imaging method has a high reproducibility of 2–4% across lipid types [13]. Although standard methods of high-performance lipid chromatography for lipid extracts [40] and optical oxygen probes for hypoxia [41] are available, these invasive methods might be more suited to cell culture and preclinical models of cancer. This study has a small cohort size, based on the standards of conventional DCE MRI. However, the novel imaging method of CSEI allows the imaging of specific biological processes, reducing confounding factors. This allows the investigation to focus on a specific target, with the possibility to develop personalised care and stratified treatment [42]. Although the primary finding was negative, it provides crucial information for the readers on the main driver of NACT responsiveness without incurring reporting bias. Targeting lipid composition and hypoxia might offer the earliest window of opportunity for intervention since deregulation of lipid composition in the peri-tumoural region of the breast [17] and hypoxia mitigation through lymphovascular invasion [7] are the central characteristics of early breast cancer initiation and aggression. However, diets rich in MUFAs and certain types of PUFAs might modulate lipid uptake and metabolism [43], while elevated hypoxia might be instantaneous [8]. This could suggest that other potential factors, such as diet, lifestyle, tumour microenvironment, and genetic factors, could impact lipid composition and hypoxia, and for trials that target dysregulated lipid metabolism [44], or dietary advice on caloric restriction [45], alternative and innovative markers of treatment response might also be required during NACT. The investigation was a prospective, registered clinical trial with recruitment of consecutive patients, and a consistent schedule for individual MRI scans ensured comparability between patients. A head-to-head comparison was carried out between good and poor responders undergoing identical treatment regimes. The treatment used in this study was in line with the guidelines in the UK during the study, and no alteration to standard clinical care was made to ensure wide applicability of the findings.

This study has limitations. First, future large cohort studies are required to confirm the findings to support decision-making in breast cancer management. This study was conducted in a focused, prospective longitudinal trial with the primary hypothesis that early response in peri-tumoural lipid composition and hypoxia might differentiate good responders from poor responders. The secondary outcome was limited to the correlation between lipid composition and hypoxia and immune response to ensure sufficient statistical power. The effects of lipid composition and hypoxia on specific treatment response, including tumour grade and size, and clinical outcomes such as quality of life, should be investigated in future studies in breast cancer patients. Second, the proportion of molecular subtypes was not controlled, although it was recorded for the purpose of the identification of outliers. Lipid deregulation might be more severe in triple-negative breast cancer (TNBC) that does not respond well to hormone or anti-HER2 therapies [46,47], and targeted evaluation in TNBC will be valuable in future studies since immunotherapy is becoming an increasingly valuable alternative for the treatment of TNBC patients [48]. Third, the spatial distribution of lipid composition was not evaluated in the small cohort. The spatial heterogeneity of lipid composition in the peri-tumoural region indicated tumour aggressiveness [13], and the spatial metrics of interaction between CD163 and cancer cells were associated with progression-free survival [26]. Although spatial metrics can be computed to provide a more comprehensive understanding of the distribution of peri-tumoural lipid composition, a larger cohort size is necessary to achieve sufficient robustness in statistics. Future large cohort studies could incorporate analysis of spatial heterogeneity to facilitate in-depth investigation of the impact on immune response in the tumour microenvironment.

## 4. Materials and Methods

### 4.1. Study Ethics

The study was approved by the London Research Ethics Committee (Identifier: 17/LO/1777) and registered as a clinical trial [NCT03501394]. Written informed consent was obtained from all participants prior to the study. 

### 4.2. Clinical Procedure

Seventeen female patients (aged 37–71 years), with grade II or III invasive ductal carcinoma and a designated treatment plan for NACT, were recruited to the study. Patients with a history of breast cancer or receiving hormonal treatment were not eligible. All patients received 5-fluorouracil 500 mg/m^2^, epirubicin 100 mg/m^2^, and cyclophosphamide 500 mg/m^2^ (FEC) once every 21 days for the first three cycles, and docetaxel 100 mg/m^2^ once every 21 days for the remaining three cycles [49,50]. Patients with HER2-positive breast cancer also received pertuzumab and trastuzumab for one year [51,52]. MRI scans were performed at 5 to 10 days (median: 7) before NACT (baseline) and 10 to 14 days (median: 12) after the first treatment cycle (Cycle1). MRI was acquired from 17 patients at baseline and 15 patients at Cycle1 (one patient was withdrawn from the study due to adverse effects from NACT and one non-compliance during scan). Standard clinical histopathological examination was performed for each patient to determine histological tumour size, grade, and nodal status for the Nottingham Prognostic Index (NPI) [53]. Immunostaining was conducted in a single batch for CD163 antibody in core biopsies and resected residual tumours with appropriate positive controls [17]. The pathological response was assessed on the resected tumours, and the poor responders and good responders were identified as below (grade 1, 2 and 3) or over (grade 4 and 5) a 90% reduction in cellularity [54]. The percentage change in CD163 was computed as the difference between resection and biopsy and normalised to biopsy.

### 4.3. Magnetic Resonance Imaging

All images were acquired on a 3 T clinical whole-body MRI scanner (Achieva TX, Philips Healthcare, Best, the Netherlands), using a 16-channel breast coil for signal detection and a body coil for uniform transmission. Patients were scanned in the prone position with the imaging volume centred on the diseased breast. Lipid composition images were acquired from all participants using a 2D CSEI sequence [55,56] with 16 echoes, an initial echo time (TE) of 1.14 ms, an echo spacing of 1.14 ms, a repetition time (TR) of 60 ms, a flip angle of 20°, a matrix size of 96 × 96, a voxel size of 2.5 mm × 2.5 mm, and a slice thickness of 5 mm. The acquisition time was 3 min and 50 s. T_2_-weighted anatomical images were acquired using a turbo spin echo sequence, with a TE of 60 ms, a TR of 5000 ms, a matrix size of 192 × 192, a voxel size of 1.25 mm × 1.25 mm, and a slice thickness of 2 mm. Diffusion-weighted images were acquired using a pulsed gradient spin echo sequence, with two *b*-values of 0 and 1000 s/mm^2^, a TE of 60 ms, a TR of 4000 ms, a matrix size of 96 × 96, a voxel size of 2.5 mm × 2.5 mm, and a slice thickness of 5 mm [17].

Image analysis was conducted in MATLAB (R2020a, Mathworks, Natrick, MA, USA) and ImageJ (v1.52p, National Institute of Health, Bethesda, MD, USA). The signal at each echo time in CSEI is the sum of the water and lipid signals, with signal decay modelled on a single transverse relaxation rate R2* [13]. The relative amplitude of individual lipid signal was used to calculate the number of double bonds in triglycerides to derive the proportion of lipid constituent, using a multi-peak spectrum model established in breast adipose tissue [13,17]. The maps of the number of double bonds in triglycerides were computed from raw data, before subsequent calculation of quantitative maps of MUFAs, PUFAs, and SFAs as a fraction of the total amount of lipids [13,17]. The map of effective transverse relaxation rate R2* was computed in the same manner as described in our previous work [13]. Tumour was delineated on the first echo of lipid composition images, with reference to T_2_-weighted anatomical and diffusion-weighted images (*b* = 1000 s/mm^2^). The peri-tumoural region, a 15 mm (6 voxels) ring surrounding the tumour boundary, was selected in accordance with common practice in the literature [17,57,58]. Although there might be a risk of an inadequate amount of peri-tumoural adipose tissue in patients with extremely dense breast, there were no patients with extremely dense breast in this study. The median of each lipid constituent and R2* in the peri-tumoural region was computed at baseline and Cycle1, with the percentage change calculated as the difference between Cycle1 and baseline and normalised to baseline.

### 4.4. Statistical Analysis

Statistical analysis was performed in R (v3.6.3, The R Foundation for Statistical Computing, Vienna, Austria). The normality of the distribution was assessed using the Shapiro–Wilk test. The difference in baseline and the percentage change in lipid composition and hypoxia between poor and good responders was compared using the Wilcoxon rank sum test. The difference in lipid composition and hypoxia between baseline and Cycle1 was compared using the Wilcoxon signed rank paired test. The correlation between lipid composition and hypoxia at baseline and CD163 in the core biopsy was performed using Spearman’s rank (*r_s_*) correlation test. The correlation between the percentage change in lipid composition and hypoxia and the percentage change in CD163 and NPI was also performed using Spearman’s test. A *p*-value < 0.05 was considered statistically significant.

## 5. Conclusions

Peri-tumoural lipid composition and hypoxia in the breast were not shown to be predictive of pathological complete response after one cycle of NACT. Treatment-induced normalisation in MUFAs and PUFAs might have potential to serve as non-invasive biomarkers for altered immune response of CD163 on TAMs and facilitate the development of novel immunotherapy agents.

## Figures and Tables

**Table 1:** Patient characteristics, tumour histology, and hormonal receptor status as grouped by the Miller–Payne system (poor responders: 1, 2, 3; good responders: 4, 5). Median (IQR) of age, tumour size, and NPI are shown.

Characteristic	All (*n* = 15)	Good Responders (*n* = 7)	Poor Responders (*n* = 8)	*p*-Value
Age	51 (43–60)	49 (38–56)	52 (47–60)	NS
Tumour size (mm, baseline)	22 (18–25)	23 (21–25)	20 (16–25)	NS
Nottingham Prognostic Index (NPI)	5.24 (4.68–6.10)	4.66 (4.03–5.05)	5.90 (5.24–6.40)	NS
*Histology*				
Invasive ductal carcinoma	15	7	8	
*Grade*				
Grade II	1	1	0	
Grade III	14	6	8	
*Hormonal receptor status*				
Oestrogen receptor positive (ER+)	6	2	4	
Human epidermal growth factor receptor 2 positive (HER2+)	2	2	0	
Triple negative (TN)	7	3	4	

NS: not significant.

**Table 2:** Comparison of lipid composition and R2* between responder groups after the first cycle of NACT and the association with CD163 and the Nottingham Prognostic Index (NPI).

Parameter	Percentage Change at Cycle1 (%)			Good vs. Poor ^a^	Percentage Change CD163 ^b^		NPI ^b^	
	All (*n* = 15)	Good Responder (*n* = 7)	Poor Responder (*n* = 8)	*z* Score, *p* Value	*r_s_, p* Value	95% CI	*r_s_, p* Value	95% CI
*Peri-tumoural*								
MUFAs	0.75 (−0.02–1.58)	0.37 (−0.15–1.09)	0.91 (0.19–2.46)	0.96, 0.336	0.59, 0.021 *	(0.11, 0.85)	−0.09, 0.790	(−0.66, 0.54)
PUFAs	4.34 (−0.06–8.12)	2.03 (−0.55–5.77)	6.13 (0.99–11.00)	0.96, 0.336	0.57, 0.027 *	(0.08, 0.84)	−0.05, 0.873	(−0.63, 0.56)
SFAs	−1.89 (−2.61–0.03)	−0.94 (−2.61–0.20)	−1.98 (−2.43–−0.49)	0.06, 0.955	−0.26, 0.354	(−0.68, 0.29)	−0.09, 0.790	(−0.66, 0.54)
R2*	−6.62 (−12.32–0.83)	−3.88 (−8.25–3.15)	−10.07 (−14.01–−6.37)	1.31, 0.189	−0.50, 0.056	(−0.81, 0.01)	−0.18, 0.593	(−0.70, 0.47)

^a^ Wilcoxon rank sum test. ^b^ Spearman’s rank correlation test. * *p* < 0.05.

## Data Availability

The raw data supporting the conclusions of this article will be made available by the authors on request.

## Supplementary Materials

The following supporting information can be downloaded at: https://www.mdpi.com/article/10.3390/ijms25179303/s1.
