# Supplementary material for: Peri-Tumoural Lipid Composition and Hypoxia for Early Immune Response to Neoadjuvant Chemotherapy in Breast Cancer
Source: Int J Mol Sci. 2024 Aug 28;25(17):9303. doi: 10.3390/ijms25179303 (PMC11395063; doi:10.3390/ijms25179303)
Supplement: Supplementary file 1 [file ijms-25-09303-s001.zip › ijms-3167486-supplementary.pdf]

Supplementary Material

Supplementary tables and figures for the clinical study

Table S1. Comparison of lipid composition and R2* between Baseline and Cycle1

Peri-tumoural monounsaturated fatty acids (MUFAs), polyunsaturated fatty acids (PUFAs), saturated fatty acids (SFAs) and R2* in the breast of patients before (baseline) and after one cycle (Cycle1) of neoadjuvant chemotherapy (n = 15). Values are presented as median and interquartile range (median (IQR)). Statistical significant difference ($p < 0.05$) is marked with an asterisk (*).

**Parameter** [a]	**Time points**		**Cycle1 vs Baseline** [b]
	**Baseline**	**Cycle1**	***z* Score, *p* Value**
Peri-tumoural			
MUFAs	0.41 (0.40 – 0.42)	0.42 (0.40 – 0.43)	1.92, 0.055
PUFAs	0.11 (0.10 – 0.13)	0.12 (0.10 – 0.14)	1.85, 0.064
SFAs	0.48 (0.45 – 0.51)	0.46 (0.43 – 0.49)	1.92, 0.055
R2*	28.86 (27.35 – 33.22)	27.44 (26.59 – 32.99)	2.36, 0.018*

[a] Unit – MUFAs, PUFAs, SFAs: fraction; R2*: s^{-1}. [b] Wilcoxon signed rank paired test.

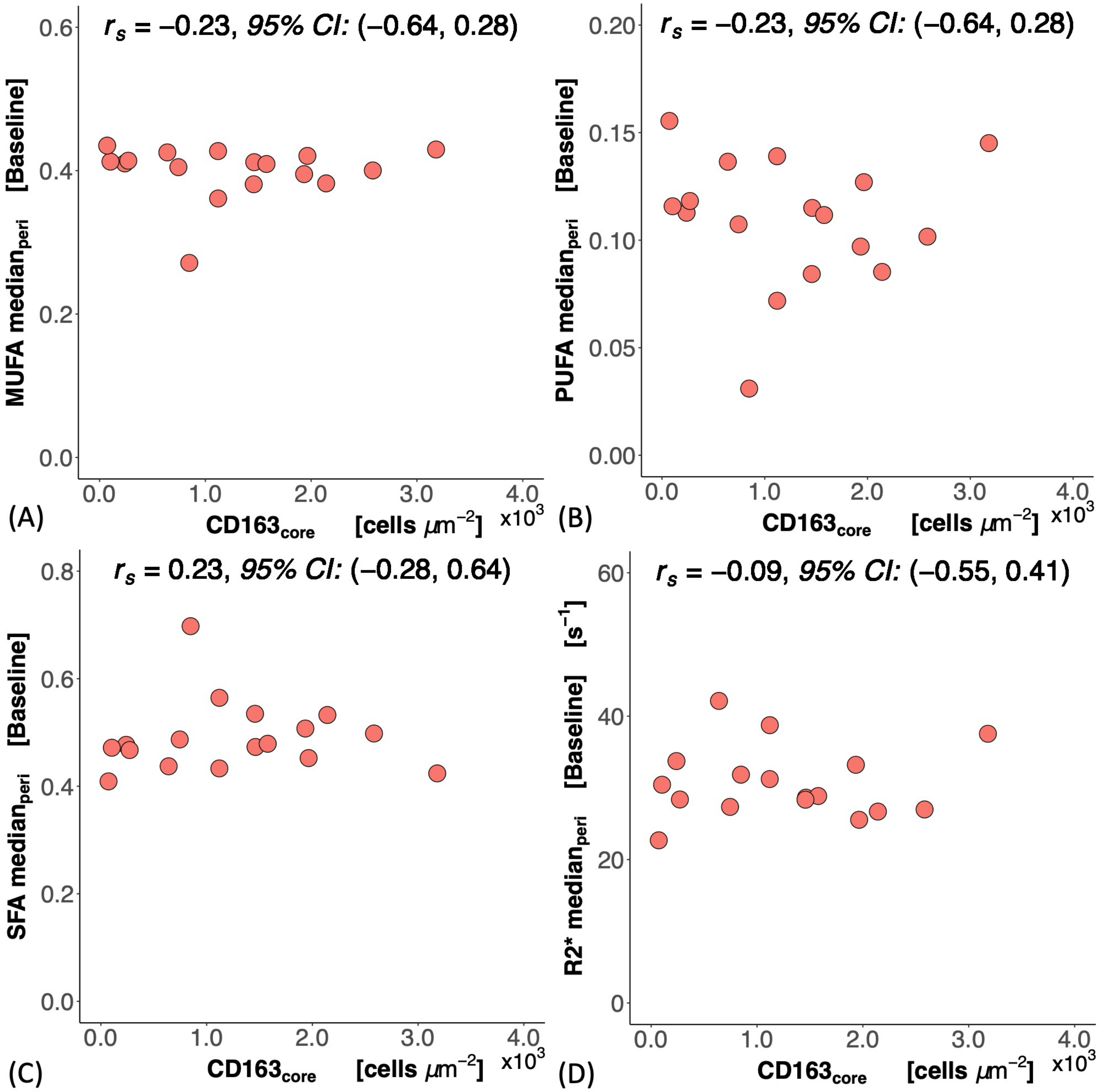

Figure S1. The correlations between peri-tumoural (**A**) monounsaturated fatty acids (MUFAs), (**B**) polyunsaturated fatty acids (PUFAs), (**C**) saturated fatty acids (SFAs) and (**D**) R2* before neoadjuvant chemotherapy (baseline) and CD163 in the core biopsy are shown in scatter plots. Spearman's rank correlation coefficient (r_s) was used for correlation analysis, and the respective 95% confidence intervals (95% CI) are shown in each plot.

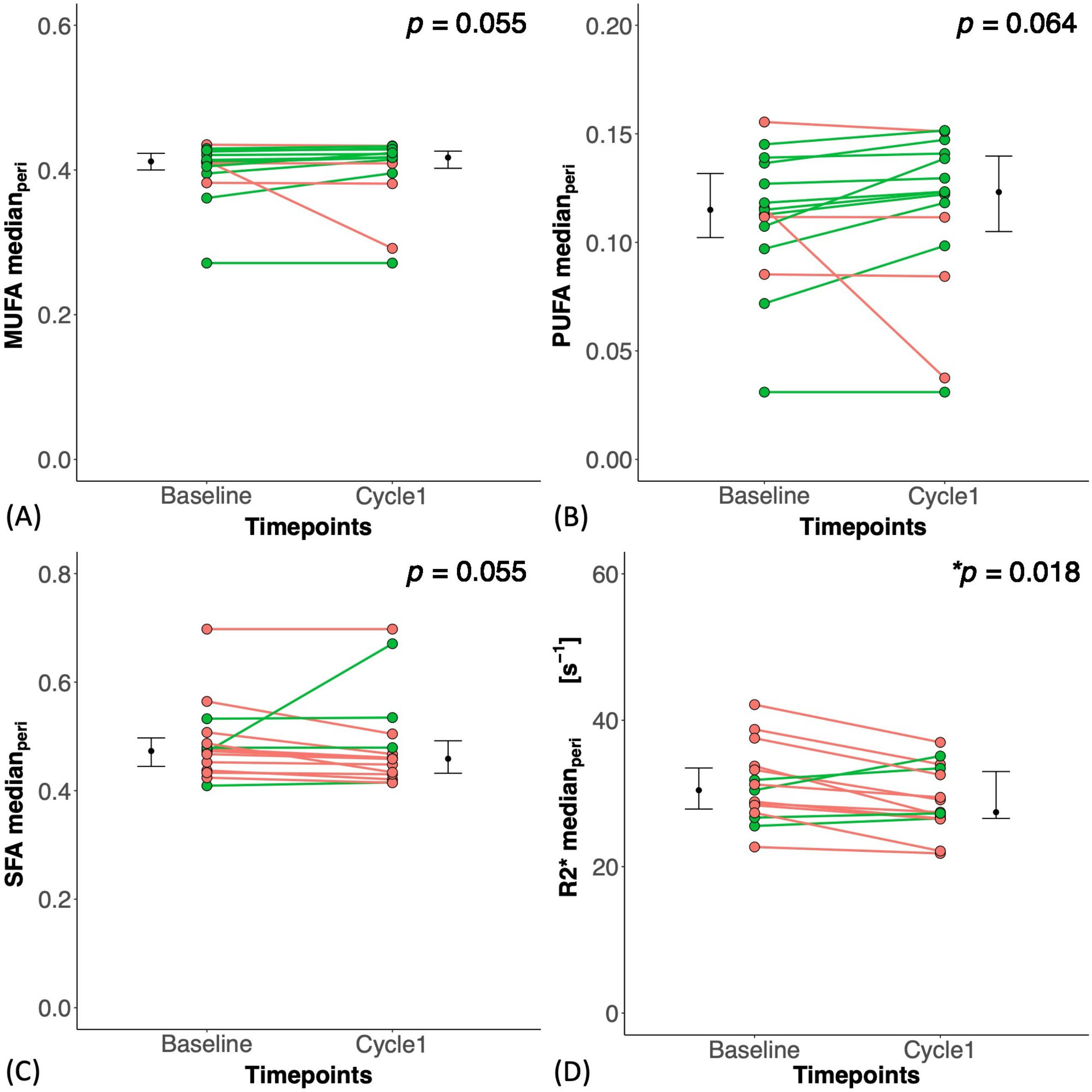

Figure S2. The longitudinal changes in peri-tumoural (**A**) monounsaturated fatty acids (MUFAs), (**B**) polyunsaturated fatty acids (PUFAs), **(C)** saturated fatty acids (SFAs) and (**D**) R2* in all participants before (baseline) and after one cycle (Cycle1) of neoadjuvant chemotherapy. Each dot represents the fraction of lipid composition or R2* in an individual patient. A red line indicates a net decrease while a green line indicates a net increase. The error bar represents the median (IQR), and *p*-values are shown in the upper right corner. Statistical significant difference ($p < 0.05$) is marked with an asterisk (*).

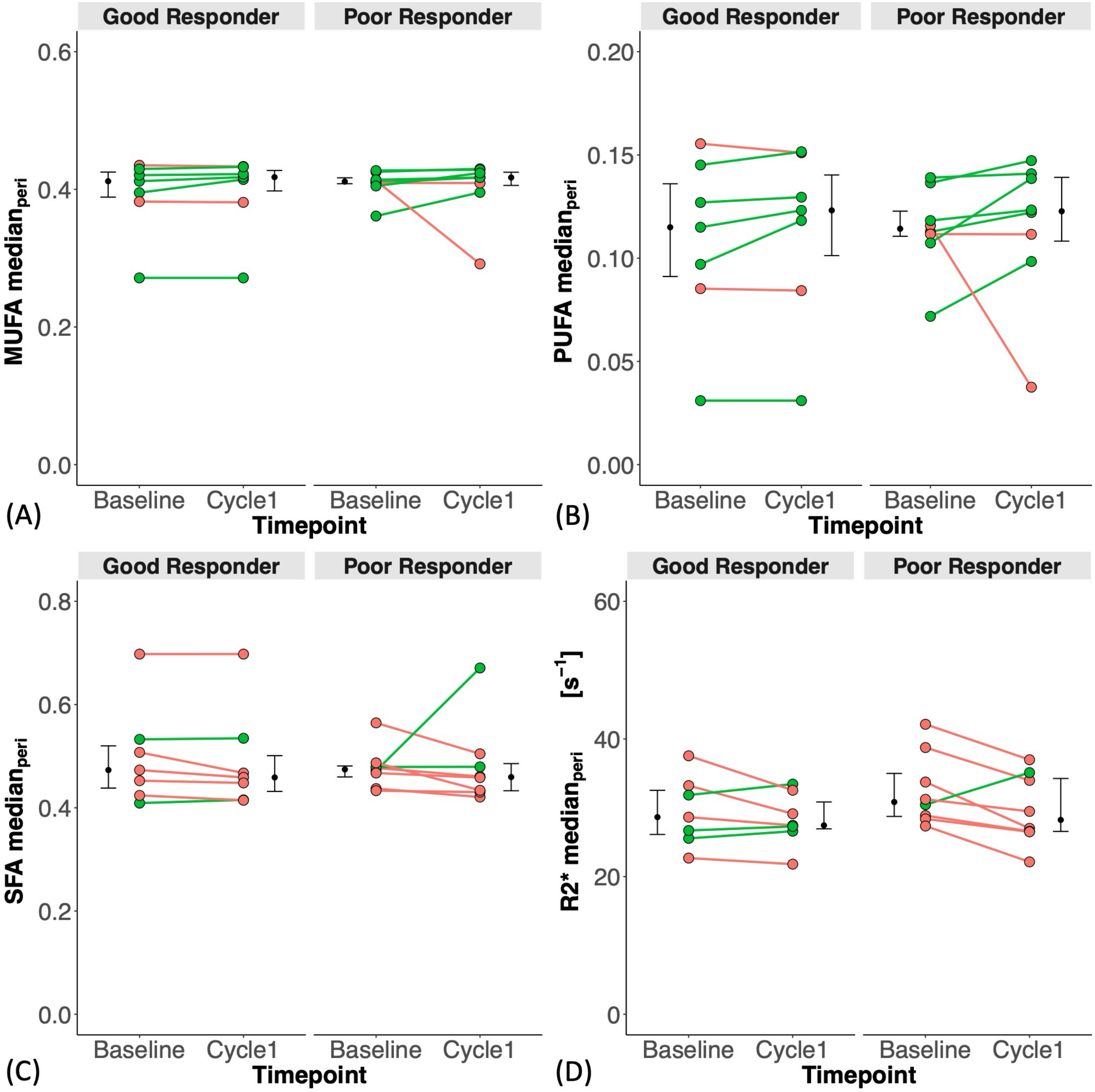

Figure S3. The longitudinal changes in peri-tumoural (**A**) monounsaturated fatty acids (MUFAs), (**B**) polyunsaturated fatty acids (PUFAs), (**C**) saturated fatty acids (SFAs) and (**D**) R2* in good and poor responders before (baseline) and after one cycle (Cycle1) of neoadjuvant chemotherapy. Each dot represents the fraction of lipid composition or R2* in an individual patient. A red line indicates a net decrease while a green line indicates a net increase. The error bar represents the median (IQR).
